# Cordycepin ameliorates morphine tolerance by inhibiting spinal cord ferroptosis and inflammation via targeting SIRT1

**DOI:** 10.7150/ijms.108518

**Published:** 2025-03-31

**Authors:** Zheng Li, Jie Liu, Jie Ju, Xiaoling Peng, Wei Zhao, Jihao Ren, Xiaoqian Jia, Jihong Wang, Feng Gao

**Affiliations:** 1 Department of Anesthesiology and Pain Medicine, Hubei Key Laboratory of Geriatric Anesthesia and Perioperative Brain Health, and Wuhan Clinical Research Center for Geriatric Anesthesia, Tongji Hospital, Tongji Medical College, Huazhong University of Science and Technology, China.; 2 Department of Anesthesiology, The First Affiliated Hospital of Shenzhen University, Shenzhen Second People's Hospital, Shenzhen, China.

**Keywords:** morphine tolerance, cordycepin, ferroptosis, inflammation, SIRT1

## Abstract

Morphine tolerance caused by long-term use of morphine is a major medical problem. Neuroinflammation plays an important role in morphine tolerance, and currently no drugs have been found for clinical use to alleviate neuroinflammation during morphine tolerance. Cordycepin is the main active component of fungus cordycepin militaris, has been demonstrated to have anti-oxidative stress and anti-inflammatory properties in various diseases. In this study, we established a rat model of morphine tolerance, examined the effect of cordycepin on the development of morphine tolerance, and evaluated its potential regulatory mechanisms. We found that cordycepin treatment ameliorated the development of morphine tolerance, improved mitochondrial damage associated with ferroptosis, by reducing the levels of reactive oxygen species (ROS), malondialdehyde (MDA) and Fe^2+^, increasing superoxide dismutase (SOD) and glutathione (GSH) levels, and decreasing the secretion of pro-inflammatory factors (IL-1β, IL-6, and TNF-α). Besides, cordycepin upregulated the expression of SIRT1, SLC7A11 and GPX4. Further research found that the above effects of cordycepin on morphine-tolerant rats were abolished by SIRT1 selective inhibitor EX-527. Thus, these findings indicated that cordycepin could ameliorate the development of morphine tolerance by inhibiting spinal cord ferroptosis and inflammation via targeting SIRT1. Collectively, these results demonstrated the protective effects of cordycepin and highlighted its therapeutic potential as a drug component for morphine tolerance treatment and prevention.

## Introduction

Morphine and its pharmacological derivatives are widely used as a potent analgesic for the management of acute and chronic pain. However, drug tolerance can occur with the long-term use of morphine, which limits its clinical utility^1^. Although pain can be relieved by increasing doses of morphine, the severity of its side effects also increases. Several factors, including neuroinflammation, oxidative stress, mitochondrial damage, apoptosis, and synaptic plasticity injury, are found to participate in the development of morphine tolerance^2^-^7^. Despite extensive research, no effective strategies have been found to prevent and reverse morphine tolerance in clinic. Therefore, to understand the mechanisms of morphine tolerance and explore the solution to solve this problem has become an important goal.

Ferroptosis is a new form of cell death characterized by iron-catalyzed aberrations in lipid peroxide metabolism, which exhibits unique morphological feature and distinct from other cell death types such as apoptosis, necrosis, autophagy in biochemical, morphological and genetical aspect^8^, ^9^. The process of ferroptosis is regulated by solute carrier family 7 member 11 (SLC7A11) and glutathione peroxidase 4 (GPX4), and driven by Fe^2+^-dependent lipid oxidation (which occurs with iron overload) and accumulation of reactive oxygen species (ROS)^10^. Increasing researches demonstrated that ferroptosis is associated with the development and progression of many diseases, including traumatic brain injury, stroke, Alzheimer's disease, perioperative neurocognitive disorders, and chronic pain^11^-^14^. However, little is known about the impact of iron induced spinal cord ferroptosis on the development of morphine tolerance. Moreover, inflammation is also an important pathological mechanism for the occurrence and progress of morphine tolerance^15^. Ferroptosis has been demonstrated to be involved in inflammatory response, and targeting ferroptosis can effectively prevent and treat inflammatory diseases^16^, ^17^. Based on the above, anti-ferroptosis and anti-inflammatory therapy may be potential strategies for alleviating morphine tolerance.

Silent information regulator 1 (SIRT1) is a kind of nicotinamide adenosine dinucleotide- (NAD-) dependent class III histone deacetylase^18^, ^19^. SIRT1 can interact with many essential transcription factors, regulate gene transcription, chromosome stability and target protein activity through deacetylation, and then participate in a series of pathophysiological processes such as oxidative stress, inflammatory, immune, autophagy, and apoptotic responses^18^, ^19^. Recent studies have shown that SIRT1 can directly or indirectly regulate SLC7A11 to enhance the ability to resist oxidative stress, thereby inhibiting ferroptosis and neuroinflammation^20^, ^21^. In addition, SIRT1 has been reported to be involved in the development of chronic pain^22^. In bone cancer induced pain models, spinal SIRT1 expression is down-regulated, while SIRT1 activator can up-regulate spinal SIRT1 expression and ameliorate bone cancer pain^23^. Also, research on the central nervous system has shown that SIRT1 plays an important role in regulation of pathogenesis of metabolic disease, neurodegenerative disorders, aging, and tumorigenesis^22^, ^24^-^26^. SIRT1 could ameliorate perioperative neurocognitive disorders in aged mice by inhibiting hippocampus ferroptosis and improve sepsis-associated encephalopathy by modulating inflammation and oxidative stress^14^, ^26^. Hence, decreased expression of SIRT1 is likely involved in the mechanism of spinal cord ferroptosis and inflammation in morphine tolerance.

Cordycepin (3'-deoxyadenosine) is the main active component of fungus cordycepin militaris. Cordycepin has a variety of biological activities, such as anti-tumor activities, anti-inflammatory activities, and anti-oxidative stress activities^27^-^29^. Numerous studies have found that cordycepin plays a pivotal role in the treatment of neurodegenerative diseases, nerve injury, and other neurological diseases by inhibiting inflammatory responses or oxidative stress^27^-^30^. Furthermore, cordycepin has been found to attenuate osteoarthritis pain through the suppression of inflammatory responses^31^. However, cordycepin has not been reported to ameliorate the development of morphine tolerance, and the underlying mechanism remains unknown.

Based on these promising findings, the present study is aimed at investigating the effects of cordycepin in the morphine-tolerant rats and determining whether cordycepin alleviates morphine tolerance by inhibiting ferroptosis and inflammation via targeting SIRT1 to activate the SLC7A11-GSH-GPX4 pathway. Our findings provide potential theoretical and experimental evidence for the treatments of morphine tolerance patients, which has significant clinical and therapeutic implications.

## Materials and methods

### Animals

Eight-week-old specific pathogen-free male Sprague-Dawley rats (body weight 220-250g) were purchased from the Laboratory Animal Center of Tongji Medical College, Huazhong University of Science and Technology (Wuhan, Hubei, China). All rats were individually housed in a room under controlled temperature and humidity conditions, with a 12/12-hour light/dark cycle, and had free access to food and water. The rats were acclimatized to the laboratory environment for 7 days before the studies. Animal handling and experimental procedures were reviewed and approved by the Ethics Committee of Tongji Medical College, Huazhong University of Science and Technology (Wuhan, Hubei, China).

### Intrathecal catheter implantation

Intrathecal catheters were implanted in the lumbar region for drug administration as previously described^32^. Briefly, the rats were deeply anesthetized with pentobarbital sodium (60 mg/kg, intraperitoneal injection (i.p.)), their lumbar and neck regions were shaved and sterilized with iodophor and ethanol. The puncture site was exposed by a 1.5-2 cm lumbar region vertical incision and then carefully and bluntly to separate the subcutaneous tissue and fascial layer with vascular forceps. Next, rats were cut the muscles above the lumbar and implanted a sterile polyethylene catheter (PE-10; outer diameter, 0.5 mm, inner diameter, 0.3 mm; Anilab Software & Instruments, Ningbo, China) into the subarachnoid cavity between L4 and L5. Subsequently, the catheter was subcutaneously tunneled, externalized, and fixed to the back of neck and the incision was closed from the muscles to the skin using sterile 4-0 sutures after disinfection with 75% (v/v) ethanol. After the operation, the rats were subcutaneous administered 10 μL normal saline to flush the catheter. Finally, they were individually placed on a heat blanket in a chamber until recovery.

### Lidocaine verification

After the third day of intrathecal catheter implantation, the correct position of the catheter was confirmed through a temporary motor block of both hind limbs. Intrathecal injection of 10 μL 2% lidocaine, followed by injection of 10 μL normal saline flushing catheter. If rats experience hind limb paralysis after injection of lidocaine, and recover within 30 minutes, it indicates that the catheter is placed correctly. Rats with hind limb paralysis after catheterization or normal hind limb muscle strength after injection of lidocaine were excluded from this study and euthanized by excessive use of pentobarbital sodium.

### Establishment of morphine tolerance models

After intrathecal catheter implantation, rats rested and recovered for 7 days. The morphine tolerance model was established using the methods introduced in previous studies^32^. Briefly, the rats were intrathecally injected with 10 µg/5 µL morphine twice daily for consecutive 7 days. Equivalent volumes of normal saline were injected at the same time points to the rats in the control group. The development of morphine tolerance was assessed by behavioral tests on days 1, 3, 5, and 7. The %MPE (see below) of morphine-injected rats gradually decreased after daily administration, indicating that the analgesic effect of morphine was weakened and the morphine tolerance model was successfully constructed.

### Experimental designs and drugs treatment

**Figure 1:**
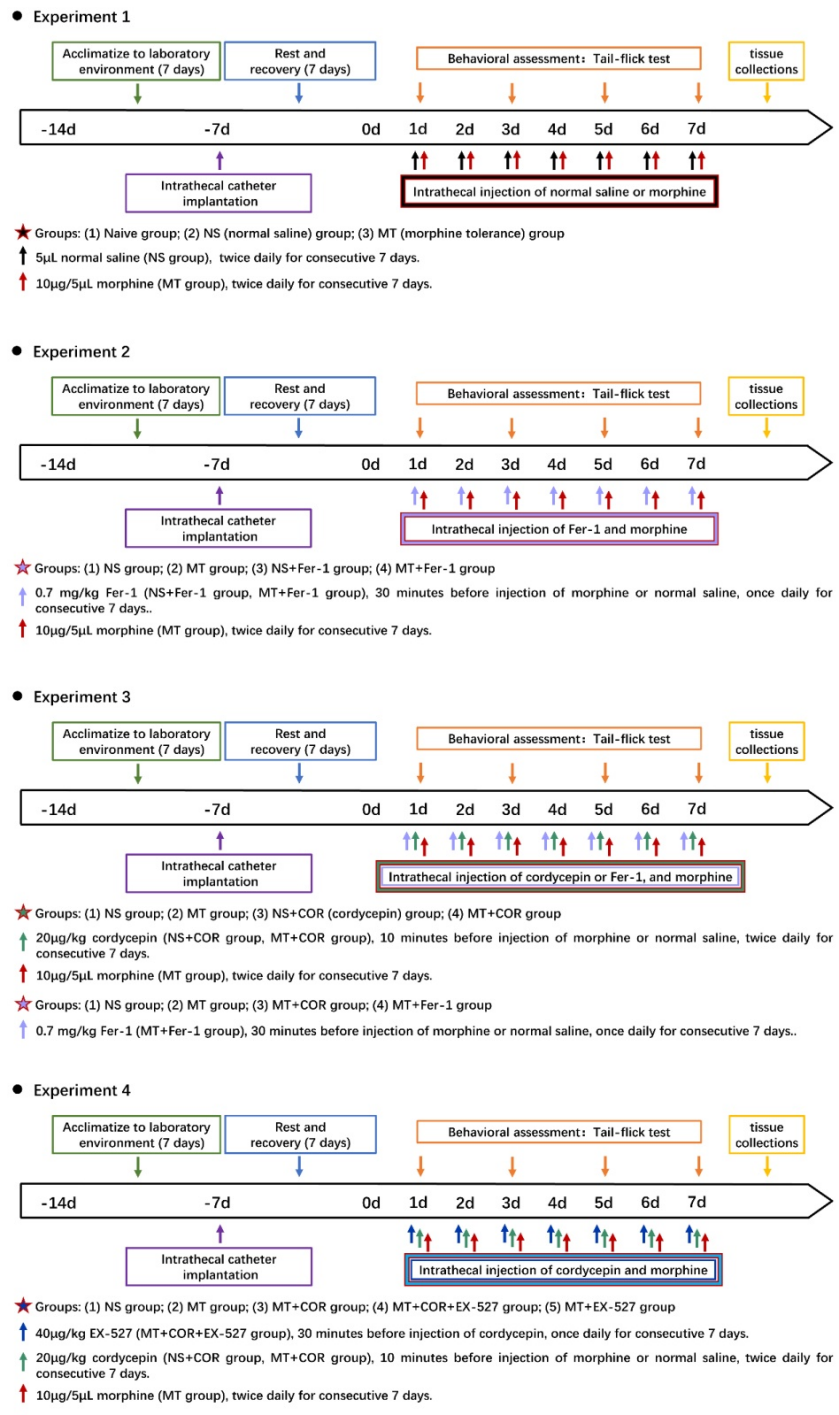
** Schematic timeline of the experimental procedure.** Experiment 1. To explore the effect of long-term use of morphine on pain threshold and the changes in SIRT1 expression in rats, the rats were intrathecally injected with morphine (10μg/5μL, twice daily, 7 days); Experiment 2. To explore the role of ferroptosis and inflammation in morphine-tolerant rats, the rats were intrathecally injected with Fer-1 (0.7 mg/kg, once daily, 7 days) 30 minutes before injection of morphine; Experiment 3. To explore the protective effect of cordycepin in morphine-tolerant rats, the rats were intrathecally injected with cordycepin (20μg/kg, twice daily, 7 days) 10 minutes before injection of morphine; Experiment 4. To explore the role of SIRT1 played on the protective effect of cordycepin in morphine-tolerant rats, the rats were intrathecally injected with EX-527 (40μg/kg, once daily, 7 days) 30 minutes before injection of cordycepin.

This study comprises four experiments, in which rats were randomly divided to different groups (All groups were n=6). And the experimental timeline was shown in **Figure 1**.

#### Experiment 1

To explore the effect of long-term use of morphine on pain threshold and the changes in SIRT1 expression in rats, the rats were randomly divided into 3 groups (n=6, per group). (1) Naive group: the rats were not subjected to any treatment; (2) NS (normal saline) group: the rats were anesthetized and received intrathecal catheter implantation. And the rats were intrathecally injected with the same volume of normal saline; (3) MT (morphine tolerance) group: the rats were anesthetized and received intrathecal catheter implantation. And the rats were intrathecally injected with morphine (10μg/5μL, twice daily, 7 days).

#### Experiment 2

To explore the role of ferroptosis and inflammation in morphine-tolerant rats, the rats were randomly divided into 4 groups (n=6, per group). (1) NS group; (2) MT group; (3) NS+Fer-1 (ferrostatin-1, a ferroptosis inhibitor) group: the rats were intrathecally injected with Fer-1 (0.7 mg/kg, once daily, 7 days, Sigma-Aldrich, MO, USA) 30 minutes before injection of normal saline; (4) MT+Fer-1 group: the rats were intrathecally injected with Fer-1 30 minutes before injection of morphine. The doses of Fer-1 were administered, as described previously^33^, ^34^.

#### Experiment 3

To explore the protective effect of cordycepin on morphine-tolerant rats, the rats were randomly divided into 4 groups (n=6, per group). (1) NS group; (2) MT group; (3) NS+COR (cordycepin) group: the rats were intrathecally injected with cordycepin (20μg/kg, twice daily, 7 days, Sigma-Aldrich, MO, USA) 10 minutes before injection of normal saline; (4) MT+COR group: the rats were intrathecally injected with cordycepin 10 minutes before injection of morphine. The doses of cordycepin were selected according to previous reports and our preliminary experiments^35^.

To explore the protective effect of cordycepin on morphine-tolerant rats is involved in inhibiting ferroptosis and inflammation, the rats were randomly divided into 4 groups (n=6, per group). (1) NS group; (2) MT group; (3) MT+COR group; (4) MT+Fer-1 group.

#### Experiment 4

To explore the role of SIRT1 played on the protective effect of cordycepin in morphine-tolerant rats, the rats were randomly divided into 5 groups (n=6, per group).(1) NS group; (2) MT group; (3) MT+COR group; (4) MT+COR+EX-527(a selective SIRT1 inhibitor): the rats were intrathecally injected with EX-527 (40μg/kg, once daily, 7 days, Selleck, Houston, TX, USA) 30 minutes before injection of cordycepin; (5) MT+EX-527 group: the rats were intrathecally injected with EX-527 30 minutes before injection of morphine. The doses of EX-527 were chosen based on earlier reports that EX-527 significantly inhibited the expression of SIRT1^36^.

### Behavioral assessment

Morphine induced analgesic tolerance was assessed by tail-flick test before drug administration and 30 minutes after morphine administration on days 1, 3, 5, and 7^2^. The detailed procedure was referred to a previous study^2^. Briefly, the rats were placed in fixed containers to restrain their bodies but not their tails. One-third of the tail was immersed in water at 50 ± 0.2°C and the time when the rats withdrew the tail (response latency) was recorded. To prevent the tail damage, the test was ended if the rats did not withdraw the tail within 15 seconds. The test was repeated thrice at a 5-minute interval between tests. And the mean of three trials was taken to be the final latency. The percentage maximal possible antinociceptive effect (%MPE) was calculated by comparing the test latency before (baseline, BL) and after (TL) drug administration using following formula: %MPE = [(TL-BL) / (15 seconds-BL)] × 100. The behavioral assessments were conducted by the experimenter who was unaware of animal grouping.

### Reactive Oxygen Species (ROS), Superoxide Dismutase (SOD), Glutathione (GSH), Malondialdehyde (MDA), and Ferrous ion (Fe^2+^) assays

The rats were decapitated under deep anesthesia, and the L3-L5 spinal cord segments were immediately collected. The fresh tissues of spinal cord were perfused with PBS containing heparin to remove blood and clots. After weighing the tissue, it was homogenized in slurry medium. The level of ROS was detected using Reactive oxygen species Assay Kit (Jiancheng Biology, Nanjing, China). The activity of SOD was detected using T-SOD Activity Assay Kit (WST-1 Method) (Elabscience, Wuhan, China). The relative concentration of GSH was detected using GSH Colorimetric Assay Kit (Elabscience, Wuhan, China). The MDA content was detected using MDA Colorimetric Assay Kit (TBA Method) (Elabscience, Wuhan, China). And the determination of Fe^2+^ level using Ferrous iron Colorimetric Assay Kit (Elabscience, Wuhan, China). All kits were used according to the manufacturer's instructions.

### Western blot analysis

The L3-L5 spinal cord segments was quickly collected under deep anesthesia and quickly bathed in a radioimmunoprecipitation assay (RIPA) lysis buffer combined with proteinase inhibitors. Subsequently, the tissue was ground into a suspension and lysed on ice for 30 minutes and then centrifuged at 12,000 rpm in a cold centrifuge (Centrifuge 5424R, rotor size: FA-45-24-11, Eppendorf, Germany) at 4°C for 15 minutes. Afterward, the supernatant was carefully transferred into new EP tube. Protein concentration was determined by BCA Protein Assay Kit (Boster, Wuhan, China). The protein lysate was diluted to the same concentration with 5×loading buffer and denatured through 10 minutes boiling. Then, the proteins (20μg) in each sample were separated by 10% sodium dodecyl sulfate-polyacrylamide gel electrophoresis (SDS-PAGE). Electrophoresis was conducted at 80 V for 40 minutes first, and then, at 120 V for 50 minutes. And subsequently transferred onto a 0.45 μm polyvinylidene fluoride (PVDF) membrane. The membranes were blocked with 5% non-fat dry milk in TBST (10 mM Tris-HCl, 150 mM NaCl, 0.1% (v/v) Tween-20, pH 7.5) at room temperature for 3 hours and then washed in TBST 5 times for 5 minutes each, and incubated overnight at 4°C with the following primary antibodies: rabbit anti- SLC7A11 (1:1,000 dilution, A2413; ABclonal), rabbit anti-GPX4 (1:1,000 dilution, A11243; ABclonal), mouse anti-SIRT1 (1:1,000 dilution, 60303-1-Ig; proteintech), mouse anti-β-actin (1:5000 dilution, AC004; ABclonal). After that, the membranes were washed 5 times with TBST for 5 minutes each time and incubated with goat anti-rabbit immunoglobulin G (IgG) (H+L) horseradish peroxidase (HRP) (1:1,000 dilution, AS014; ABclonal), or goat anti-mouse IgG (H+L) HRP (1:3,000 dilution, AS003; ABclonal) at room temperature for 1.5 hours. The protein bands were visualized with SuperLumia enhanced chemiluminescence (ECL) detection reagents (Abbkine, USA) and a computerized image analysis system (Bio-Rad, ChemiDoc XRS+, USA). Image J software was used to quantify protein blot intensity.

### RNA isolation and quantitative real-time polymerase chain reaction (qRT-PCR) assays

The L3-L5 spinal cord segments was quickly collected under deep anesthesia. Total RNA was extracted from the spinal cord tissue with TRIzol reagent (Thermo Fisher Scientific, Shanghai, China) according to the manufacturer's instructions. Isolated RNA was then reverse transcribed into cDNA using the HiScript® II RT SuperMix (Vazyme, Nanjing, China) following the standard protocol. For real-time quantitative PCR analysis, the resultant cDNA products were amplified using a 2×ChamQ Universal SYBR qPCR Master Mix (Vazyme, Nanjing, China) in triplicate. The following primers (Servicebio, Wuhan, China) were used in the process: rat IL-1β (forward 5'- ATA GCA GCT TTC GAC AGT GAG G -3'; reverse 5'- GGA GAA TAC CAC TTG TTG GCT TA -3'), rat IL-6 (forward 5'- CAG CCA CTG CCT TCC CTA CTT C -3'; reverse 5'- TAG CCA CTC CTT CTG TGA CTC TAA CT -3'), rat TNF-α (forward 5'- CGT CGT AGC AAA CCA CCA AGC -3'; reverse 5'- CCA GTC GCC TCA CAG AGC AAT -3'), rat GAPDH (forward 5'-CGC TAA CAT CAA ATG GGG TG-3'; reverse 5'- TGC TGA CAA TCT TGA GGG AG-3'). The housekeeping gene GAPDH was the internal control. The data were normalized to GAPDH and relative expressions of genes were calculated by the 2^-ΔΔCt^ method^2^.

### Immunofluorescence assays

The rats were perfused with saline followed by 4% cold paraformaldehyde under deep anesthesia, and the L3-L5 spinal cord segments were excised and washed with PBS and then fixed at 4°C for 24 hours. The tissues were embedded in paraffin, and the spinal cord were sliced into 5 μm slices and placed on adhesive slides and dewaxed thoroughly. Tissue sections were treated with 0.3% (v/v) Triton X-100 and then blocked with 10% (v/v) donkey serum at room temperature for 1 hour. After washing, the sections were incubated at 4°C overnight with the following primary antibodies: mouse anti-SIRT1 (1:100 dilution, 60303-1-Ig; proteintech), goat anti-Iba1 (1:100, ab5076, Abcam). Finally, the sections were incubated with the secondary antibodies CoraLite594-conjugated Donkey Anti-Mouse IgG(H+L) (1:200, SA00013-7, proteintech), or Fluorescein (FITC)-conjugated Donkey Anti-Goat IgG(H+L) (1:100, SA00003-3, proteintech) at room temperature for 2 hours and stained with 4,6-diamidino-2-phenylindole (DAPI) at room temperature for 10 minutes. Fluorescent images were captured under a fluorescence microscope (Olympus, Tokyo, Japan).

### Transmission electron microscope (TEM)

In order to study the morphology of mitochondria in the spinal cord, the rats were decapitated while under anesthetized, the L3-L5 spinal cord segments were quickly separated and cut into 1 mm^3^ pieces with a sharp scalpel, and soaked immediately in 2.5% cold glutaraldehyde. Next, these tissues were fixed, dehydrated, embedded, solidified, sectioned, and stained. Finally, the ultrastructural characteristics of spinal cord mitochondria were observed under the TEM (HT7800, HITACHI, Japan).

### Statistical analysis

Data were analyzed using GraphPad Prism software (GraphPad Software, San Diego, CA, USA). All data were presented as mean ± standard error of the mean (SEM). Group differences in behavioral assessment were analyzed using the two-way analysis of variance (ANOVA) with Bonferroni post hoc test. Others statistical significance was analyzed using one-way ANOVA followed by Bonferroni post hoc test. P<0.05 was considered to be statistically significant.

## Results

### Long-term morphine administration induces drug tolerance

**Figure 2:**
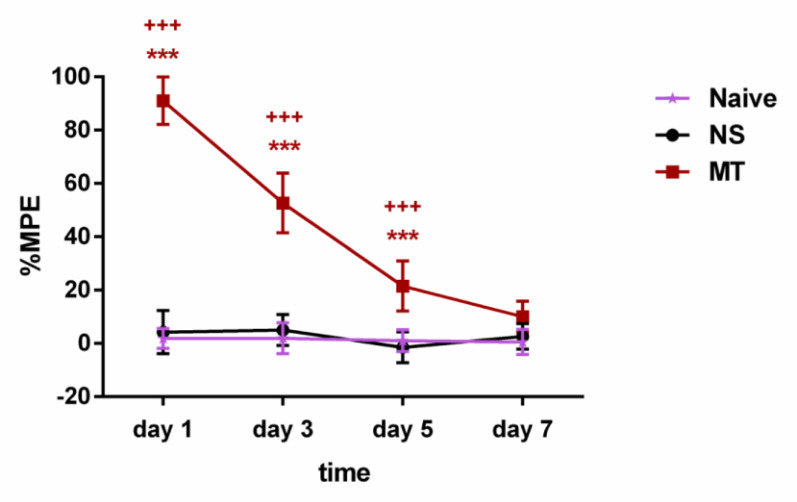
** Long-term morphine administration induces drug tolerance.** The rats were randomly divided into 3 groups: Naive group, NS group, and MT group. Thermal pain threshold was assessed using percentage maximal possible antinociceptive effect (%MPE) based on rat tail-flick latency. Data are expressed as mean ± SEM (n = 6/group). ***P < 0.001, NS vs. MT; ^+++^P < 0.001, Naive vs. MT. NS: normal saline; MT: morphine tolerance.

To verify the reliability of the model, we evaluated the influence of chronic morphine administration in rats using the tail-flick test. The rats were intrathecally administered with 10µg/5µL morphine twice daily for consecutive 7 days. The tail-flick test was conducted before and 30 minutes after morphine administration on days 1, 3, 5, and 7. As shown in **Figure 2**, the rats received morphine exhibited significantly higher %MPE when compared with the NS group rats on days 1, 3, and 5 of morphine administration. However, on day 7, there was no significant differences in %MPE level between the MT group and NS group rats. There was no significant difference in %MPE level between the Naive group and NS group during the 7-day observation period. Hence, our results suggested that the rats have developed morphine tolerance on day 7.

### Ferroptosis and inflammation are involved in morphine tolerance

Ferroptosis and inflammation, are hot topics in recent research, were reported to exist in various neurological disease. Therefore, we speculated that ferroptosis and inflammation might play important roles in the development of morphine tolerance. To confirm this, we use ferroptosis inhibitor Fer-1 and collected the L3-L5 spinal cord of rats to test the key indicators of ferroptosis and inflammation.

**Figure 3:**
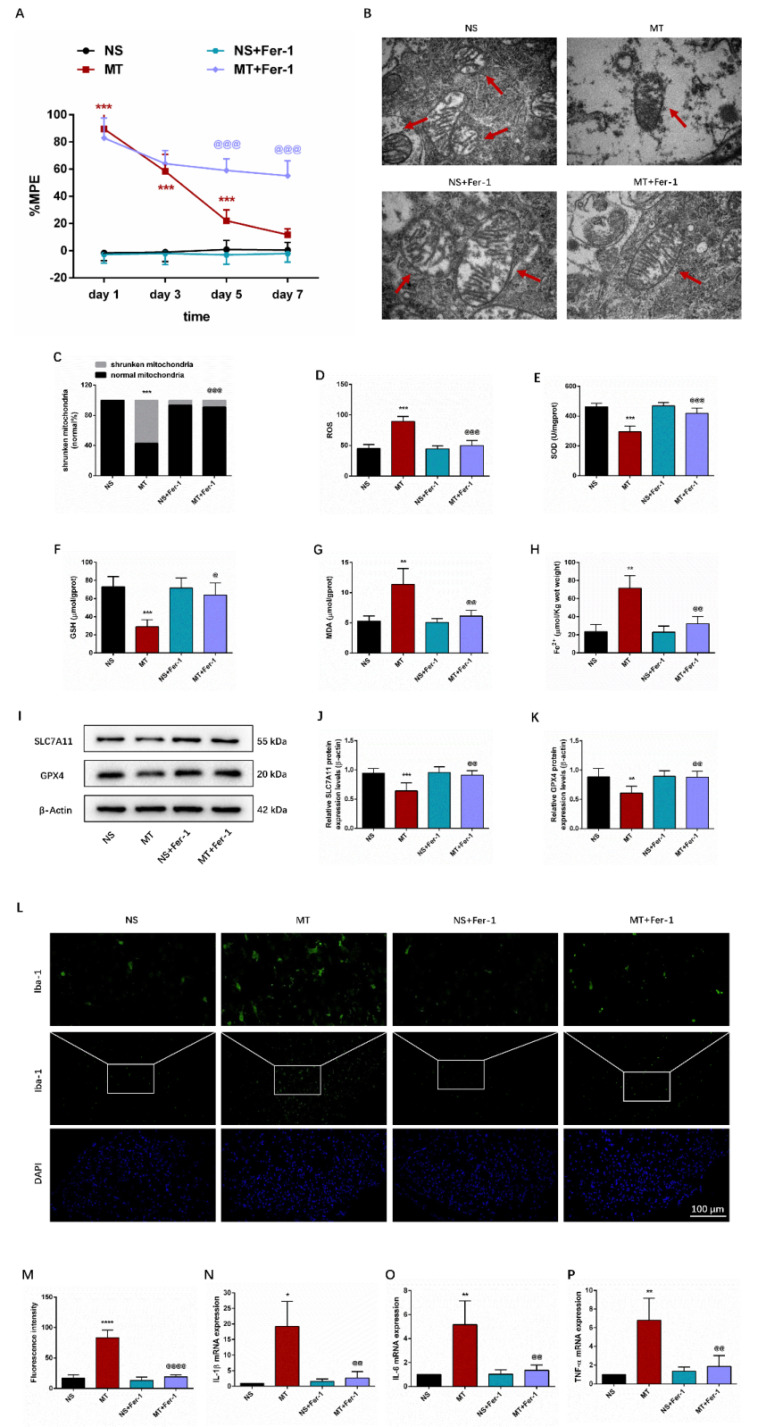
** Ferroptosis and inflammation are involved in morphine tolerance.** The rats were randomly divided into 4 groups: NS group, MT group, NS+Fer-1 group, and MT+Fer-1 group. A. Thermal pain threshold was assessed using percentage maximal possible antinociceptive effect (%MPE) based on rat tail-flick latency; B. Transmission electron microscopy (TEM) was employed to detect the ultrastructure of spinal cord in rats (Bar =500 nm); C. Shrunken mitochondrial frequency; D. The ROS level by ROS Assay Kit; E. The SOD level by T-SOD Activity Assay Kit; F. The GSH level by GSH Colorimetric Assay Kit; G. The MDA level by MDA Colorimetric Assay Kit; H. The Fe^2+^ level by Ferrous iron Colorimetric Assay Kit; I-K. SLC7A11 and GPX4 expression in each group were determined by Western blot; L. Representative images of immunofluorescence staining of Iba-1 (Bar =100 μm); M. Quantification analysis of Iba-1; N-P. The levels of IL-1β, IL-6, and TNF-α in each group were determined by qRT-PCR. Data are expressed as mean ± SEM (n = 6/group). *P < 0.05, **P < 0.01, ***P < 0.001, ****P < 0.0001, NS vs. MT; ^@^P < 0.05, ^@@^P < 0.01, ^@@@^P < 0.001, ^@@@@^P < 0.0001, MT vs. MT+Fer-1. NS: normal saline; MT: morphine tolerance; Fer-1: ferrostatin-1, a ferroptosis inhibitor.

Behavioral tests showed that compared with the MT group, the %MPE from day 5 to 7 was significantly higher in the MT+Fer-1 group **(Figure 3A)**. And there was no significant difference between the NS group and NS+Fer-1 group.

Ferroptosis results in morphological alterations in mitochondrial ultrastructure. TEM analysis revealed that mitochondria morphology of the spinal cord in the MT group showed the significant characteristic changes of ferroptosis, including the volume of mitochondrial became smaller, the density of double-layer membrane was increased, and the mitochondrial crest was decreased** (Figure 3B-3C)**.

We then examined ferroptosis-related oxidative stress, Fe^2+^-dependent lipid peroxidation, and key ferroptosis regulation-related protein SLC7A11 and GPX4. As shown in **Figures 3E-3H**, we observed that compared with the NS group, SOD and GSH levels in the MT group were lower, while ROS, MDA and Fe^2+^ levels were higher. Meanwhile, the protein expression levels of SLC7A11 and GPX4 were decreased in the MT group (**Figures 3I-3K**). Besides, Fer-1 treatment could reverse these changes (**Figure 3D-3K**).

In addition, we examined inflammatory markers in the spinal cord through immunofluorescence staining and qRT-PCR. We found that the activity of Iba-1 and the secretion of pro-inflammatory factors IL-1β, IL-6, and TNF-α were significantly increased in the spinal cord of morphine-tolerant rats, and these changes were significantly restored after Fer-1 treatment (**Figures 3L-3P**).

Taken together, these data suggested that ferroptosis and inflammation may be associated with the development of morphine tolerance.

### Cordycepin treatment alleviates the development of morphine tolerance

**Figure 4:**
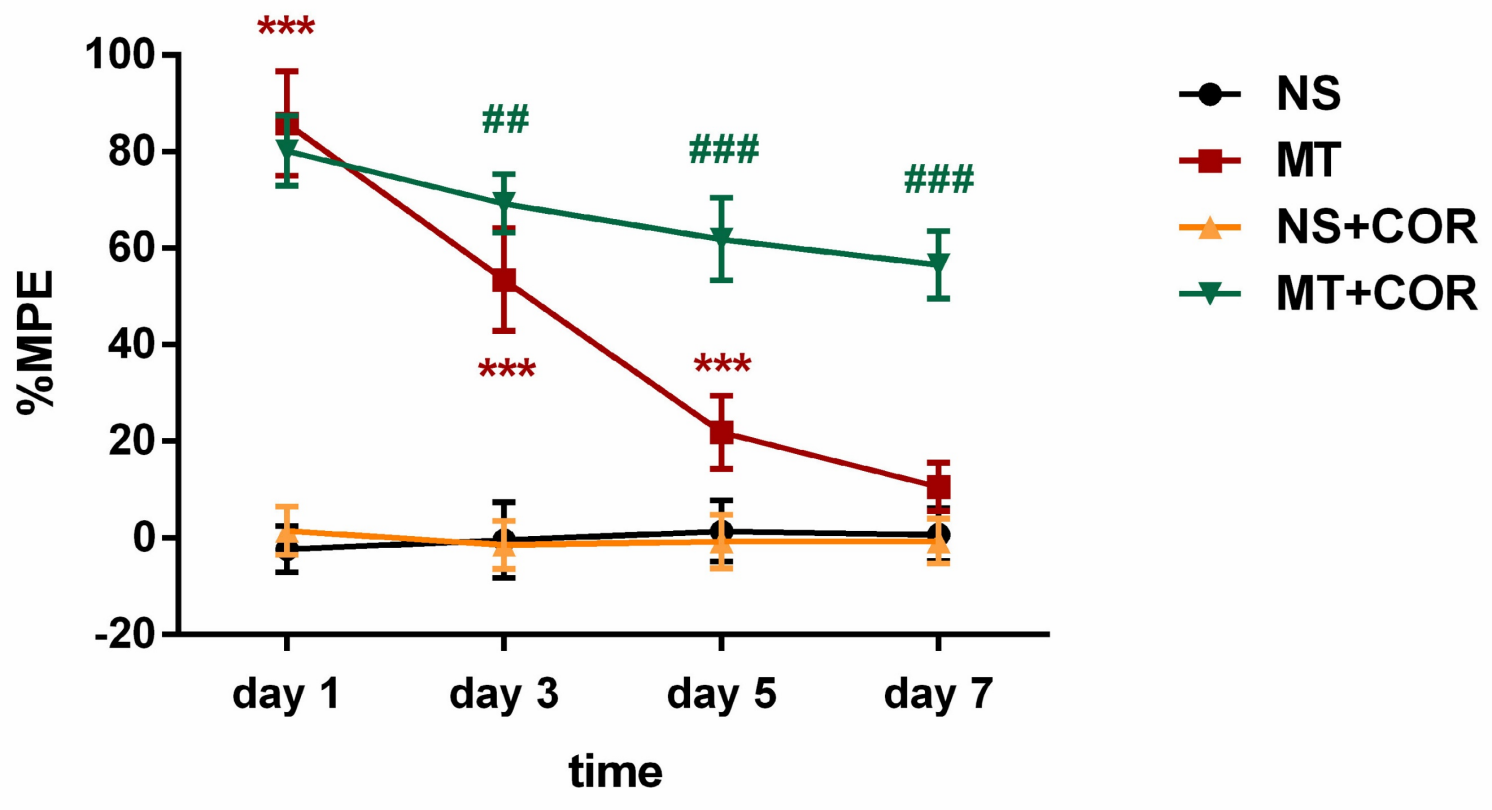
** Cordycepin treatment alleviates the development of morphine tolerance.** The rats were randomly divided into 4 groups: NS group, MT group, NS+COR group, and MT+COR group. Thermal pain threshold was assessed using percentage maximal possible antinociceptive effect (%MPE) based on rat tail-flick latency. Data are expressed as mean ± SEM (n = 6/group). ***P < 0.001, NS vs. MT; ^##^P < 0.01, ^###^P < 0.001, MT vs. MT+COR. NS: normal saline; MT: morphine tolerance; COR: cordycepin.

The %MPE of rats were evaluated through behavioral experiments after administration of cordycepin. The results suggested that the MT group exhibited significantly higher %MPE than the NS group rats on days 1, 3, and 5 of morphine administration, but, on day 7, there was no significant differences in %MPE level between the MT group and NS group rats (**Figure 4**). In contrast, the %MPE from day 3 to 7 in MT+COR group was significantly higher than those in MT group (**Figure 4**). There was no difference between the NS group and the NS+COR group. These results indicated that COR treatment can alleviate the development of morphine tolerance.

### Cordycepin inhibits ferroptosis and inflammation in the spinal cord of morphine-tolerant rats

**Figure 5:**
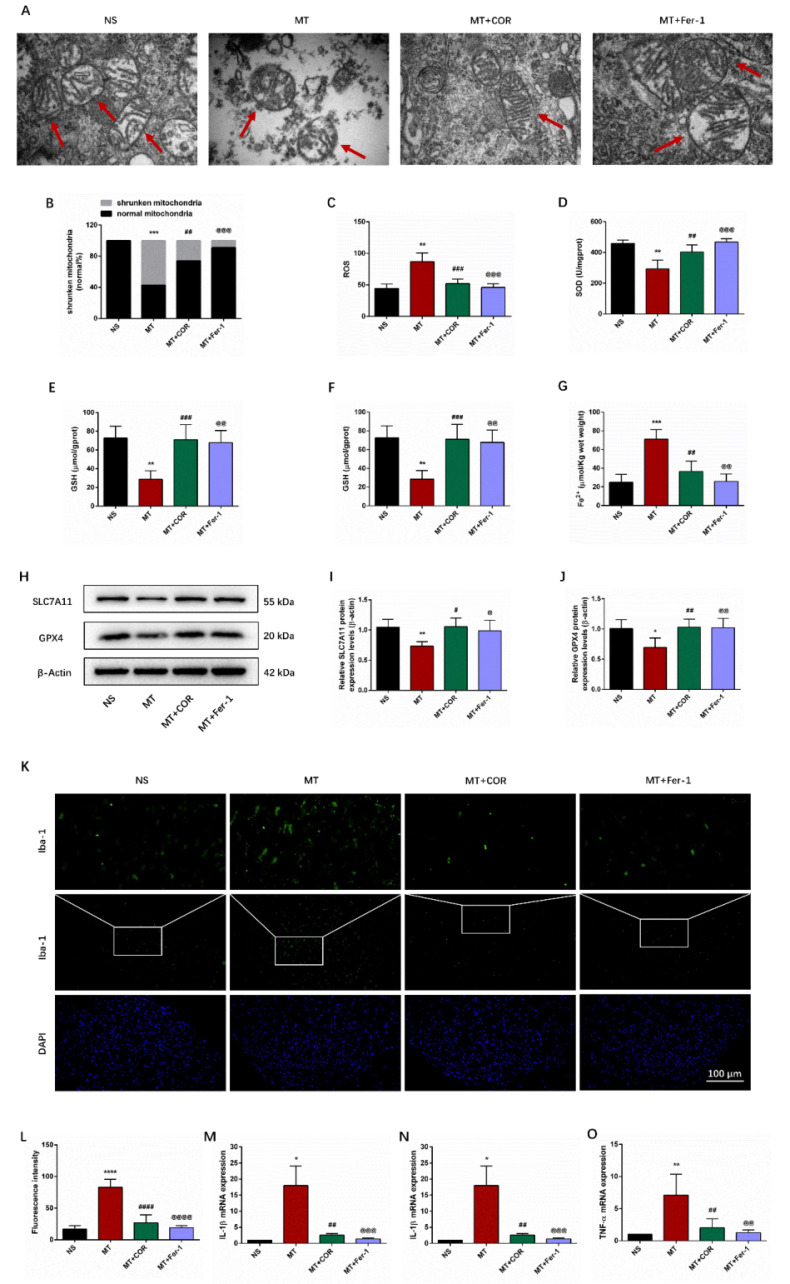
** Cordycepin inhibits ferroptosis and inflammation in the spinal cord of morphine-tolerant rats.** The rats were randomly divided into 4 groups: NS group, MT group, MT+COR group, and MT+Fer-1 group. A. Transmission electron microscopy (TEM) was employed to detect the ultrastructure of spinal cord in rats (Bar =500 nm); B. Shrunken mitochondrial frequency; C. The ROS level by ROS Assay Kit; D. The SOD level by T-SOD Activity Assay Kit; E. The GSH level by GSH Colorimetric Assay Kit; F. The MDA level by MDA Colorimetric Assay Kit; G. The Fe^2+^ level by Ferrous iron Colorimetric Assay Kit; H-J. SLC7A11 and GPX4 expression in each group were determined by Western blot; K. Representative images of immunofluorescence staining of Iba-1 (Bar =100 μm); L. Quantification analysis of Iba-1; M-O. The levels of IL-1β, IL-6, and TNF-α in each group were determined by qRT-PCR. Data are expressed as mean ± SEM (n = 6/group). *P < 0.05, **P < 0.01, ***P < 0.001, ****P < 0.0001, NS vs. MT; ^#^P < 0.05, ^##^P < 0.01, ^###^P < 0.001, ^####^P < 0.0001, MT vs. MT+COR; ^@^P < 0.05, ^@@^P < 0.01, ^@@@^P < 0.001,^ @@@@^P < 0.0001, MT vs. MT+Fer-1. NS: normal saline; MT: morphine tolerance; COR: cordycepin; Fer-1: ferrostatin-1, a ferroptosis inhibitor.

We use Fer-1 to further determine whether the protective effect of cordycepin on morphine tolerance is involved in inhibiting ferroptosis and inflammation. TEM analysis revealed that compared with the MT group, the volume of mitochondria was increased, the density of double-layer membrane was reduced, and the mitochondrial crest was increased in the MT+COR group and MT+Fer-1 group **(Figure 5A-5B)**. Additionally, compared with increased ferroptosis in MT group, significantly reduced ROS, MDA, and Fe^2+^ levels combined with elevated SOD and GSH levels was found in the MT+COR group and MT+Fer-1 group **(Figures 5C-5G)**. Meanwhile, the results of Western blot showed that the protein expression levels of SLC7A11 and GPX4 were increased in the MT+COR group and MT+Fer-1 group compared with the MT group **(Figures 5H-5J)**. As shown in** Figures 5K-5O**, compared with the MT group, the activity of Iba-1 and the mRNA levels of IL-1β, IL-6, and TNF-α in the MT+COR group and MT+Fer-1 group were significantly attenuated. These findings suggested that cordycepin attenuates morphine tolerance by inhibiting spinal cord ferroptosis and inflammation.

### Cordycepin alleviates the development of morphine tolerance and spinal cord ferroptosis and inflammation of morphine-tolerant rats in a SIRT1-dependent manner

**Figure 6:**
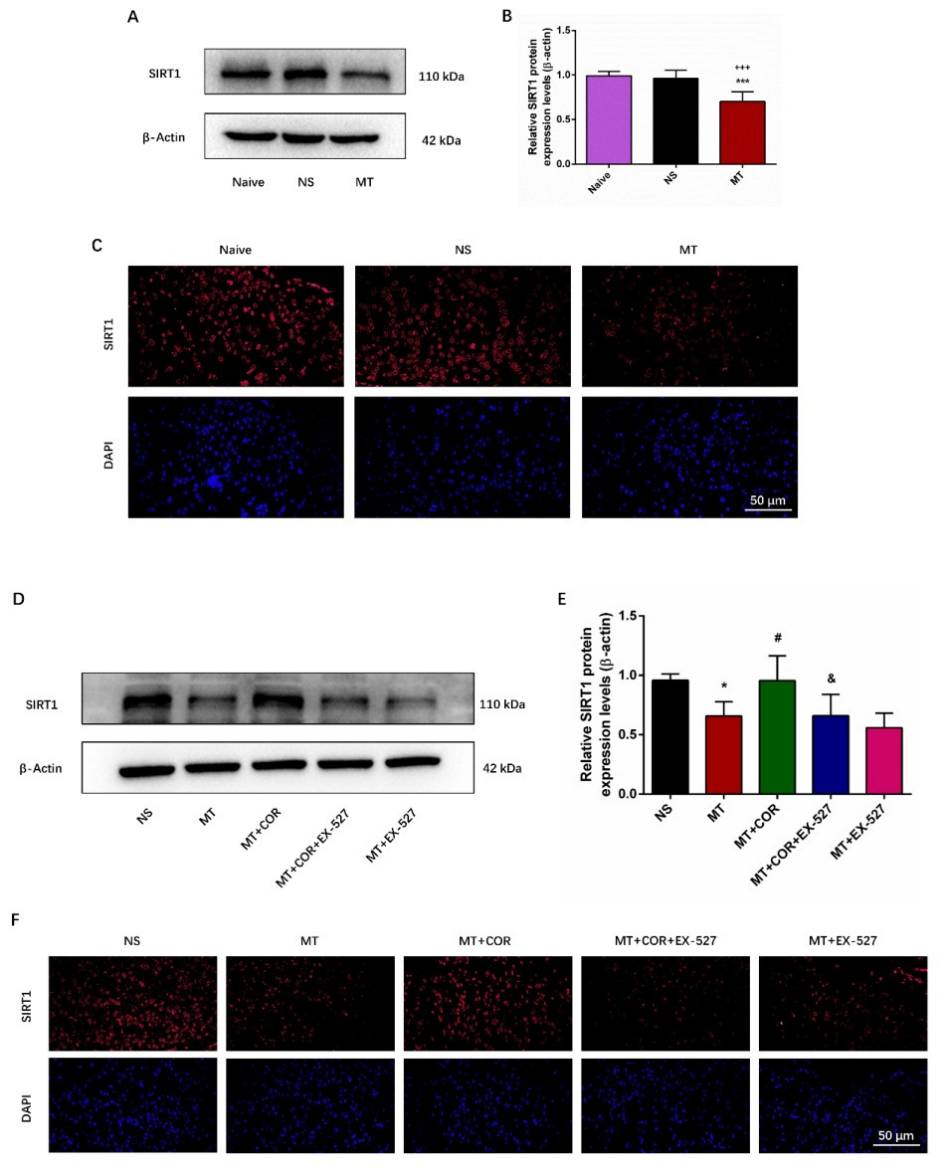
** Cordycepin alleviates the development of morphine tolerance in a SIRT1-dependent manner.** The rats were randomly divided into 5 groups: NS group, MT group, MT+COR group, MT+COR+EX-527 group, and MT+EX-527. A-B. SIRT1 expression in each group were determined by Western blot; C. Representative images of immunofluorescence staining of SIRT1 (Bar =50 μm); D-E. SIRT1 expression in each group were determined by Western blot; F. Representative images of immunofluorescence staining of SIRT1 (Bar =50 μm). Data are expressed as mean ± SEM (n = 6/group). *P < 0.05, ***P < 0.001, NS vs. MT; ^#^P < 0.05, MT vs. MT+COR; ^&^P < 0.05, MT+COR vs. MT+COR+EX-527. NS: normal saline; MT: morphine tolerance; COR: cordycepin; EX-527: a specific SIRT1 inhibitor.

SIRT1 has been found to play a crucial role in regulating oxidative stress and inflammatory response^22^. Notably, our study found that repeated morphine administration decreased the protein expression of SIRT1 **(Figures 6A-6B)**. And the immunofluorescence staining outcomes showed that the fluorescence signal of SIRT1 (red) in MT group was significantly weakened **(Figures 6C)**. There was no difference between the Naive group and the NS group. To explore whether the effect of cordycepin is SIRT1 dependent, we treated rats with EX-527, a specific SIRT1 inhibitor. The data of Figure **6D-6F** showed that cordycepin can up-regulate the expression level of SIRT1, but, EX-527 eliminates this effect of cordycepin. These results indicated that the effect of cordycepin was mediated by regulation of SIRT1.

**Figure 7:**
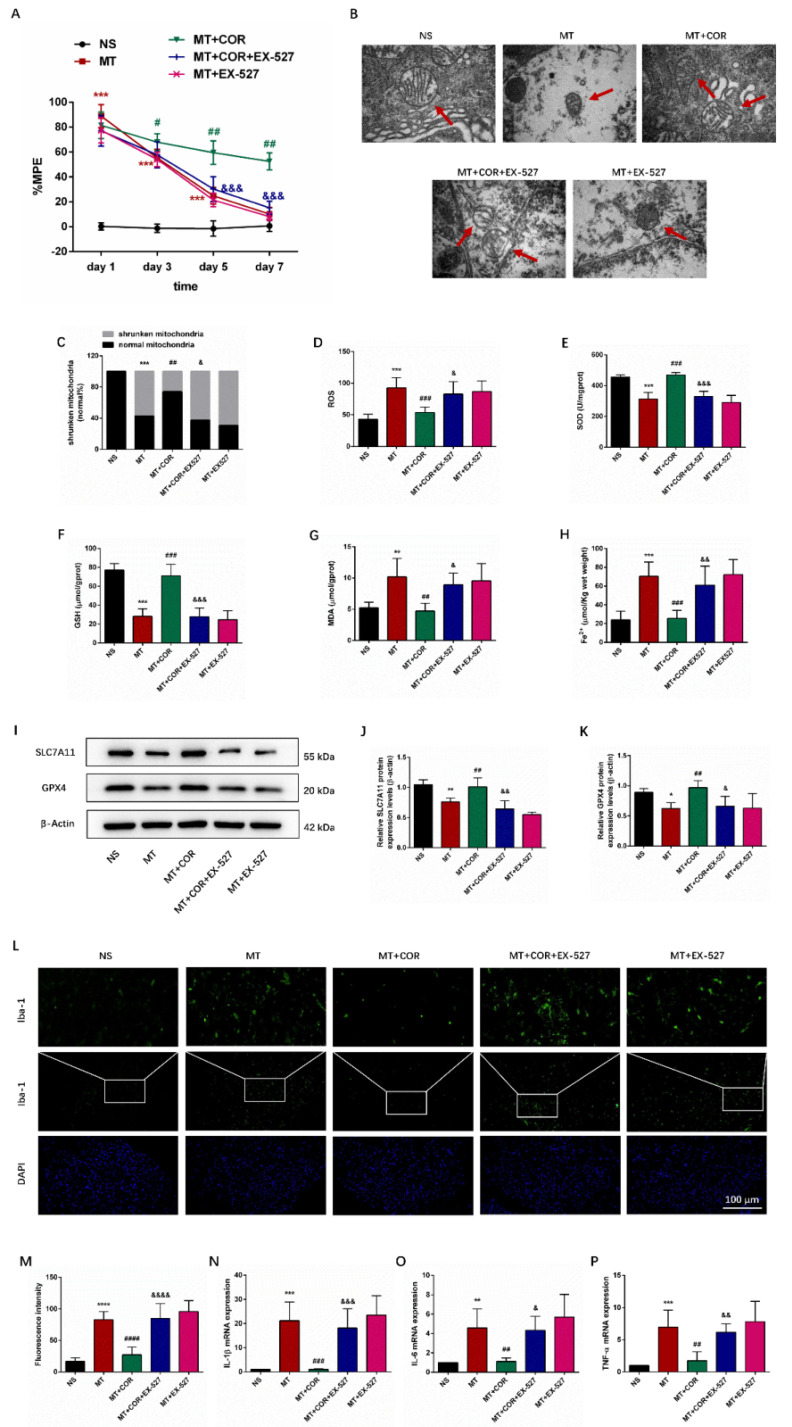
** Cordycepin alleviates spinal cord ferroptosis and inflammation of morphine-tolerant rats in a SIRT1-dependent manner.** The rats were randomly divided into 5 groups: NS group, MT group, MT+COR group, MT+COR+EX-527 group, and MT+EX-527. A. Thermal pain threshold was assessed using percentage maximal possible antinociceptive effect (%MPE) based on rat tail-flick latency; B. Transmission electron microscopy (TEM) was employed to detect the ultrastructure of spinal cord in rats (Bar =500 nm); C. Shrunken mitochondrial frequency; D. The ROS level by ROS Assay Kit; E. The SOD level by T-SOD Activity Assay Kit; F. The GSH level by GSH Colorimetric Assay Kit; G. The MDA level by MDA Colorimetric Assay Kit; H. The Fe^2+^ level by Ferrous iron Colorimetric Assay Kit; I-K. SLC7A11 and GPX4 expression in each group were determined by Western blot; L. Representative images of immunofluorescence staining of Iba-1 (Bar =100 μm); M. Quantification analysis of Iba-1; N-P. The levels of IL-1β, IL-6, and TNF-α in each group were determined by qRT-PCR. Data are expressed as mean ± SEM (n = 6/group). *P < 0.05, **P < 0.01, ***P < 0.001, ****P < 0.0001, NS vs. MT; ^#^P < 0.05,^ ##^P < 0.01,^ ###^P < 0.001, ^####^P < 0.0001, MT vs. MT+COR; ^&^P < 0.05, ^&&^P < 0.01,^ &&&^P < 0.001,^ &&&&^P < 0.0001, MT+COR vs. MT+COR+EX-527. NS: normal saline; MT: morphine tolerance; COR: cordycepin; EX-527: a specific SIRT1 inhibitor.

Additionally, behavioral tests results suggested that the MT group and MT+EX-527 group exhibited significantly higher %MPE than the NS group rats on days 1, 3, and 5 of morphine administration, but, on day 7, there was no significant differences in %MPE level between them **(Figure 7A)**. In contrast, the %MPE from day 3 to 7 in MT+COR group was significantly increased **(Figure 7A)**. Interestingly, the use of EX-527 abolished the protective effects of cordycepin on morphine tolerance. Compared with the MT+COR group, the %MPE from day 3 to 7 was significantly decreased in MT+COR+EX-527 group. These data indicated that cordycepin treatment alleviates the development of morphine tolerance in a SIRT1-dependent manner.

We then examined the effect of cordycepin-mediated SIRT1 activation on spinal cord ferroptosis and inflammation in morphine-tolerant rats. The results showed that cordycepin alleviated spinal cord ferroptosis and inflammation of morphine-tolerant rats were abolished by EX-527 administration. Compared with the NS group, the mitochondria in MT group and MT+EX-527 group had shrunk in volume, the double-layer membrane density had increased, and the mitochondrial crest had decreased or disappeared. In contrast, cordycepin ameliorated ferroptosis-induced mitochondrial morphologic changes, and treating MT+COR mice with EX-527 the mitochondria morphology in spinal cord showed the characteristic changes of ferroptosis **(Figure 7B-7C)**. In addition, compared with the NS group, the SOD and GSH levels were lower, while ROS, MDA, and Fe^2+^ levels were higher in the MT group and MT+EX-527 group **(Figures 7D-7H)**. Meanwhile, the protein expression levels of SLC7A11 and GPX4 were decreased **(Figures 7I-7K)**. There was no difference between the MT group and MT+EX-527 group **(Figures 7B-7K)**. In contrast, cordycepin treatment increased the levels of SOD, GSH, GPX4, and SLC7A11 and decreased the levels of ROS, MDA and Fe^2+^ which were reversed by EX-527 administration **(Figures 7B-7K)**. Furthermore, the immunofluorescence staining and qRT-PCR results revealed that compared with the MT group, cordycepin treatment decreased the activity of Iba-1 and the mRNA levels of IL-1β, IL-6, and TNF-α in the spinal cord, whereas EX-527 administration strongly abolished these regulatory effects of cordycepin **(Figures 7L-7P)**. These data suggested that cordycepin inhibits spinal cord ferroptosis and inflammation in morphine-tolerant rats by targeting SIRT1 to activate the SLC7A11-GSH-GPX4 pathway.

Taken together, our results suggested that cordycepin effectively ameliorated the development of morphine tolerance and inhibited spinal cord ferroptosis and inflammation through a SIRT1-dependent mechanism.

## Discussion

**Figure 8:**
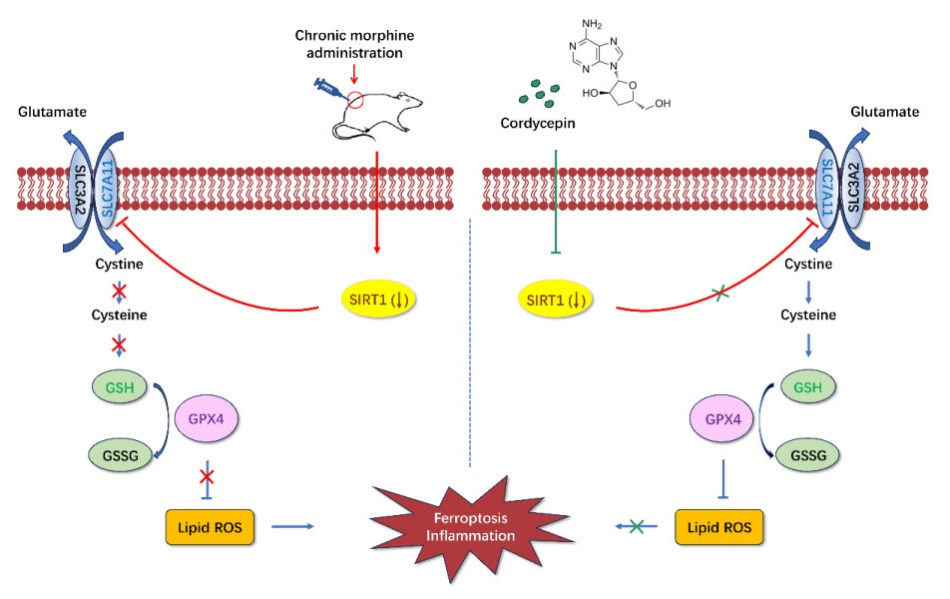
** Diagrammatic presentation of the findings from this study.** Long-term use of morphine downregulates SIRT1 and leads to spinal cord ferroptosis and inflammation, which in turn leads to drug tolerance. Cordycepin upregulates the expression level of SIRT1. Activation of SIRT1 inhibits spinal cord ferroptosis and inflammation. SIRT1: silent information regulator 1; SLC7A11, carrier family 7 membrane 11; GSH, glutathione; ROS, reactive oxygen species; GPX4, glutathione peroxidase 4.

The current study was conducted to examine the effects and mechanisms of cordycepin on chronic morphine induced analgesic tolerance in rats. Here, we found that long-term morphine administration induced drug tolerance and spinal cord ferroptosis and inflammation. Cordycepin could alleviate the development of morphine tolerance by inhibiting ferroptosis and inflammation. In addition, cordycepin could reverse the expression level of SIRT1 reduced by long-term morphine administration. And administration specific SIRT1 inhibitor EX-527 abolished improving effects of cordycepin in morphine-tolerant rats. The protective effects of cordycepin on the development of morphine tolerance were related to its inhibited spinal cord ferroptosis and inflammation by targeting SIRT1 to activate the SLC7A11-GSH-GPX4 pathway (**Figure 8**).

Iron is one of the most important minerals, which plays an indispensable role in many physiological and pathological processes of the body^37^. In central nervous system, iron homeostasis is important for maintaining body normal physiological functions, including enzyme catalysis, mitochondrial function, myelination, and synaptic plasticity. Imbalance of iron homeostasis can cause oxidative stress and inflammation, leading to cell damage and ultimately leading to neurological disease^16^, ^38^, ^39^. Ferroptosis is a programmed cell death process associated with dysregulation of iron homeostasis, which is characterized by iron dependent lipid peroxidation^40^. Previous studies suggested that ferroptosis was present in cardiovascular disease and neuropathic pain in rats^41^, ^42^. And ferroptosis was considered as a target for the treatment of inflammatory diseases, such as secondary brain injury (intracerebral hemorrhage), acute kidney injury, and spinal cord injury^43^-^46^. In this experiment, we found the increased Fe^2+^ level in spinal cord of morphine-tolerant rats. Meanwhile, the levels of ROS, MDA, IL-1β, IL-6, and TNF-α were increased, while the levels of SOD, GSH, SLC7A11, and GPX4 were decreased. In addition, treatment with Fer-1 reversed these changes, indicating that ferroptosis and inflammation contribute to the development of morphine tolerance.

Cordycepin, a natural nucleoside analogue isolated from culture broth of Cordyceps militaris, has an essential function in inhibiting inflammation and preventing oxidative stress^47^, ^48^. Importantly, cordycepin has been shown to attenuate obesity-related oxidative stress and enhances antioxidant capacity in mice^49^, ^50^. It has also been shown to prevent rat renal ischemia/reperfusion injury through regulating inflammation and oxidative stress^50^. Cordycepin represents a promising agent for future potential clinical application. In our study, we identified that cordycepin could downregulate the levels of ROS, MDA, Fe^2+^, IL-1β, IL-6, and TNF-α, while can increase the levels of SOD, GSH, SLC7A11, and GPX4 in morphine-tolerant rats. These results indicated that cordycepin could alleviate the development of morphine tolerance by inhibiting spinal cord ferroptosis and inflammation.

SIRT1, a NAD-dependent deacetylase, can participate in a wide variety of biological processes including oxidative stress, inflammatory response, autophagy, immune, apoptosis, and metabolism^51^-^55^. Therefore, SIRT1 may become a new target for the treatment and prevention of morphine tolerance. In this study, we found that SIRT1 expression was downregulated in the morphine-tolerant rats. Previous studies found that cordycepin could activate SIRT1 to regulate oxidative stress and ameliorates diabetes-induced testicular damage^28^. Furthermore, another study suggested that SIRT1 was activated by cordycepin to exert an anti-fatigue effect^29^. Our results found that cordycepin could restore the downregulation of SIRT1, suggesting the regulatory role of cordycepin in SIRT1 function in morphine tolerance.

In addition, our results also found that inhibiting SIRT1 activation could result in spinal cord ferroptosis and inflammation in morphine-tolerant rats. Activating SIRT1 can prevent renal fibrosis in patients with nephrolithiasis by inhibiting ferroptosis^56^. Researchers revealed that activating SIRT1 can reduce iron accumulation in lung epithelial cells and ameliorate acute lung injury^57^. It is well-recognized that ferroptosis is mainly regulated by the competition between the ferroptosis execution system and the antioxidant defense system. The SLC7A11-GSH-GPX4 signaling axis plays a central antioxidant role in resistance to ferroptosis^20^, ^58^, ^59^. Liu et al. revealed that SIRT1 abolished ischemia-reperfusion-induced ferroptosis and neuroinflammation and ameliorated brain damage by regulating SLC7A11-GSH-GPX4 pathway^21^. Our study found that the expressions of SIRT1, SLC7A11, GSH, and GPX4 were restored after cordycepin treatment, and SIRT1 inhibitor could inhibit this SLC7A11-GSH-GPX4 pathway activation, suggesting that SIRT1 could inhibit ferroptosis and inflammation by activating SLC7A11-GSH-GPX4 pathway. These results demonstrated that cordycepin effectively ameliorated the development of morphine tolerance by targeting SIRT1 to activate the SLC7A11-GSH-GPX4 pathway and inhibiting spinal cord ferroptosis and inflammation.

Nevertheless, there is a limitation in this paper. The observation of this study is drug-induced tolerance. And %MPE was used to investigate analgesic effect of morphine, but this test did not exclude the influence of drug-induced hyperalgesia^60^. In future studies, more comprehensive testing methods, such as dose-response relationships before and after morphine injections, should be adopted to explore the mechanisms of morphine tolerance.

## Conclusion

In summary, we observed that long-term use of morphine reduces the expression of SIRT1 and induces spinal cord ferroptosis and inflammation, leading to drug tolerance. Cordycepin alleviates the development of morphine tolerance by inhibiting spinal cord ferroptosis and inflammation via targeting SIRT1 to activate the SLC7A11-GSH-GPX4 pathway. Our study provides new insights into the therapeutic potential of cordycepin, and indicates that it may be a highly promising candidate for the treatment of drug tolerance.

## Abbreviations

SIRT1: Silent information regulator 1
SLC7A11: solute carrier family 7 member 11
GPX4: glutathione peroxidase 4
ROS: reactive oxygen species
NAD-: nicotinamide adenosine dinucleotide-
%MPE: percentage maximal possible antinociceptive effect
NS: normal saline
MT: morphine tolerance
Fer-1: ferrostatin-1
COR: cordycepin
SOD: superoxide dismutase
GSH: glutathione
MDA: malondialdehyde
Fe^2+^: Ferrous ion
TEM: transmission electron microscope
RIPA: radioimmunoprecipitation assay
SDS-PAGE: sodium dodecyl sulfate-polyacrylamide gel electrophoresis
PVDF: polyvinylidene fluoride
IgG: immunoglobulin G
HRP: horseradish peroxidase
qRT-PCR: quantitative real-time polymerase chain reaction
FITC: Fluorescein
DAPI: 4,6-diamidino-2-phenylindole
SEM: standard error of the mean
ANOVA: analysis of variance
